# Use of hip- versus wrist-based actigraphy for assessing functional decline and disease progression in patients with motor neuron disease

**DOI:** 10.1007/s00415-023-11584-7

**Published:** 2023-02-06

**Authors:** Cory J. Holdom, Jordi W. J. van Unnik, Ruben P. A. van Eijk, Leonard H. van den Berg, Robert D. Henderson, Shyuan T. Ngo, Frederik J. Steyn

**Affiliations:** 1 Australian Institute for Bioengineering and Nanotechnology, The University of Queensland, Brisbane, Australia; 2 Department of Neurology, UMC Utrecht Brain Center, University Medical Center Utrecht, Utrecht, The Netherlands; 3 Biostatistics and Research Support, Julius Center for Health Sciences and Primary Care, University Medical Center Utrecht, Utrecht, The Netherlands; 4 Centre for Clinical Research, The University of Queensland, Brisbane, Australia; 5 Department of Neurology, Royal Brisbane and Women’s Hospital, Herston, Australia; 6 Wesley Medical Research, The Wesley Hospital, Brisbane, Australia; 7 School of Biomedical Sciences, Faculty of Medicine, The University of Queensland, Brisbane, Australia

**Keywords:** Accelerometry, Amyotrophic lateral sclerosis, Longitudinal cohort study, Motor neuron disease

## Abstract

**Background:**

Actigraphy has been proposed as a measure for tracking functional decline and disease progression in patients with Motor Neuron Disease (MND). There is, however, little evidence to show that wrist-based actigraphy measures correlate with functional decline, and no consensus on how best to implement actigraphy. We report on the use of wrist actigraphy to show decreased activity in patients compared to controls, and compared the utility of wrist- and hip-based actigraphy for assessing functional decline in patients with MND.

**Methods:**

In this multi-cohort, multi-centre, natural history study, wrist- and hip-based actigraphy were assessed in 139 patients with MND (wrist, *n = *97; hip, *n = *42) and 56 non-neurological control participants (wrist, *n = *56). For patients with MND, longitudinal measures were contrasted with clinical outcomes commonly used to define functional decline.

**Results:**

Patients with MND have reduced wrist-based actigraphy scores when compared to controls (median differences: prop. active = − 0.053 [− 0.075, − 0.026], variation axis 1 = − 0.073 [− 0.112, − 0.021]). When comparing wrist- and hip-based measures, hip-based accelerometery had stronger correlations with disease progression (prop. active: *τ* = 0.20 vs 0.12; variation axis 1: *τ* = 0.33 vs 0.23), whereas baseline wrist-based accelerometery was better related with future decline in fine-motor function (τ = 0.14–0.23 vs 0.06–0.16).

**Conclusions:**

Actigraphy outcomes measured from the wrist are more variable than from the hip and present differing sensitivity to specific functional outcomes. Outcomes and analysis should be carefully constructed to maximise benefit, should wrist-worn devices be used for at-home monitoring of disease progression in patients with MND.

## Introduction

Motor neuron disease (MND) is a group of neurodegenerative diseases characterised by the progressive and irreversible loss of motor neurons in the brain and/or spinal cord [1]. Symptoms include a combination of wasting, stiffness, and paralysis in the affected regions. Patients have a median prognosis of 3–5 years; however, there is considerable variability in onset, progression, and survival [2]. Given the lack of effective treatments for MND, supportive care is offered to ease disease burden and to help slow disease progression [1].

Improved patient-centric measures are emerging as part of evolving care practices aimed at improving quality of life for patients with MND, as well as providing novel endpoints for clinical trials. This has resulted in a transition towards at-home patient monitoring, where the introduction of digital health care technology could benefit the delivery of care and development of treatments [3]. While generally focussed on the implementation or use of telemedicine, there has been a growing emphasis on adopting at-home measures that can provide information about disease progression, including the use of wearable sensors aimed at monitoring physical function in patients with MND. The use of such devices as a measure of disease progression has been validated to some degree [4]; however, consensus on wear time, feature extraction, and device positioning is yet to be established.

Several approaches have been reported for monitoring physical activity in MND. One clinical trial (NCT02447952) showed that a free-living, chest-worn, accelerometer effectively correlated time spent “active” with ALSFRS-R [5]. An earlier study incorporating the use of free-living hip-worn accelerometers in patients with MND found that simple activity summaries correlate with ALSFRS-R and can be used to reduce sample size for long-duration clinical trials [4]. A study (NCT03016897) evaluating the performance of devices, including a wrist accelerometer, for at-home monitoring of MND found that such devices improved patients’ perceived control over their disease [6]. Collectively, these outcomes suggest that accelerometry can monitor MND outside traditional clinical settings. Despite these promising early findings, device placement could impact patient compliance and the quality of data collected [7], and clinical utility can only be realised if metrics offer improved understanding of disease progression and outcomes.

We compare the use of hip- versus wrist-based actigraphy in monitoring functional decline in patients with MND. Results show that wrist-based actigraphy can be used to monitor progressive physical decline; however, measures are highly variable and may offer less granularity in conventional outcome measures when compared to hip-based actigraphy.

## Materials and methods

### Study approach

**Fig. 1:**
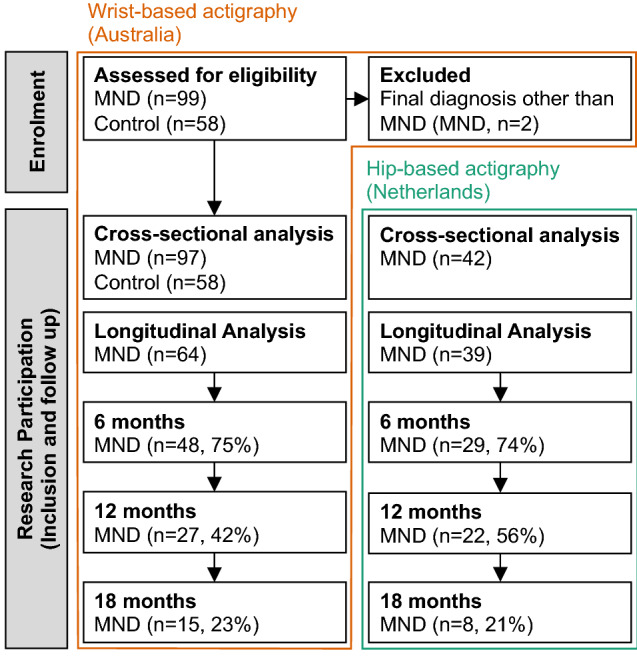
Study design. Schematic summarising the number of individuals from the Australian (wrist-worn trackers) and Dutch (hip-worn trackers) cohorts during enrolment, at study inclusion (cross-sectional analysis), and during follow-up (longitudinal analysis)

This prospective case–control multi-cohort study was conducted between February 28, 2017 and June 5, 2021 at the Royal Brisbane and Women’s Hospital (RBWH, Brisbane, Australia), and University Medical Centre Utrecht (UMCU, Utrecht, The Netherlands). Patients who received a diagnosis of MND at the RBWH MND clinic or UMCU were invited to participate (detailed in Fig. 1). Diagnosis was determined with the revised El Escorial criteria (6). Ninety-seven patients from the RBWH Motor Neurone Disease (MND) clinic and 42 patients from UMC with MND were assessed for eligibility and included in this study. Information on patient demographics and disease onset was collected at enrolment.

Enrolment and inclusion of patients from UMC were described previously [4]; 42 patients with MND completed hip-based assessments. For longitudinal analysis, 39 patients provided two or more follow-up measures. Repeat assessments were completed at three-month intervals for up to 19.7 months (median latency between assessments was 2.7 ± 1.7 months); total follow-up duration was 478.2 person-months with a median duration of 13.9 ± 11.9 months per patient. For the Australian patient cohort (wrist-based assessments), 64 patients completed two or more measures. Repeat assessments were completed at three- to four-month intervals for up to 32.2 months (median latency between assessments was 3.4 ± 1.3 months); total follow-up duration was 812.5 person-months with a median duration of 10.4 ± 10.8 months per patient. For case–control comparisons with wrist-based actigraphy, 58 non-neurodegenerative disease control participants were recruited as a sample of convenience of partners and friends of patients with MND. Considered exclusion criteria were respiratory impairment where forced vital capacity (% of GLI prediction [8]) was 60% or less at assessment for eligibility. No participants presented with this additional criterion. Compliance was calculated as the proportion of days with valid actigraphy measures (reporting days) relative to the number of days that patients were instructed to wear devices (recording days). When considering participants with MND, compliance for hip- vs. wrist-based actigraphy was 93% (694 reporting days of 746 recording days) vs. 86% (2280 reporting days out of 2648 recording days), respectively. For non-MND control participants, this was 94% (489 reporting days out of 520 recording days).

Demographics for all participants for cross-sectional analysis and for patients who completed two or more assessments (longitudinal analysis) are presented in Table 1. This study was approved by the University of Queensland and the RBWH human research ethics committees (Australia, HREC/14/QRBW/495), and the medical ethics committee and institutional board of UMCU (The Netherlands, 16/606). All participants provided written, informed consent.

**Table 1:** Demographics and measures of activity in patients with MND or controls for cross-sectional analysis, and patients with MND that progressed to longitudinal analysis

	Cross-sectional analysis						Longitudinal Analysis		
	Wrist				Hip		Wrist	Hip	
	Control (*n = *58)	MND (*n = *97)	*p*	*p*^a^	MND (*n = *42)	*p*^b^	MND (*n = *64)	MND (*n = *39)	*p*^a^
Demographics									
Age, years	55.33 ± 16.11	60.69 ± 12.55	**0.011**		61.28 ± 15.74	0.968	60.38 ± 13.70	61.69 ± 15	0.568
Female (%)	29 (50)	22 (23)	**0.001**		11 (26)	0.829	15 (24)	10 (26)	1.000
Body mass index, kg·m^−2^	26.02 ± 7.17	26.54 ± 26.54	0.722		24.20 ± 3.50	**0.013**	25.38 ± 5.28	24.10 ± 3.40	0.090
Clinical Measures									
ALS		87 (93)			39 (93)		57 (91)	36 (92)	
PLS		6 (6)			3 (7)		5 (8)	0 (0)	
PMA		1 (1)			0 (0)		1 (2)	3 (8)	
Bulbar onset (%)		21 (25)			7 (17)	0.366	14 (24)	7 (18)	0.802
Time since onset, months		21.31 ± 13.27			24.92 ± 21.39	0.100	20.90 ± 12.29	24.38 ± 22	0.120
Diagnostic delay, months		12 ± 11			7.53 ± 11.69	0.061	11.50 ± 10	8.64 ± 15	0.200
Riluzole use (%)		47 (50)			30 (75)	**0.008**	31 (49)	29 (76)	**0.012**
ALSFRS-R		38 ± 9			38 ± 12	0.937	39.50 ± 7	38 ± 12	0.858
ΔFRS, month^−1^		− 0.39 ± 0.42			− 0.34 ± 0.54	0.160	− 0.37 ± 0.34	− 0.33 ± 0.4	**0.049**
Activity Summaries									
Follow-up duration, months							10.4 ± 10.8	13.9 ± 11.9	0.666
Interval between measures, months							3.43 ± 1.33	2.73 ± 1.67	** < 0.001**
Proportion active (PA)	0.71 ± 0.10	0.65 ± 0.10	** < 0.001**	**0.011**	0.28 ± 0.14	** < 0.001**			
Vector magnitude (VM)	11.95 ± 1.28	10.36 ± 1.53	** < 0.001**	**0.003**	8.70 ± 2.97	** < 0.001**			
Variation in Axis 1 (VA1)	2.59 ± 0.13	2.52 ± 0.17	** < 0.001**	0.662	1.71 ± 0.49	** < 0.001**			
Variation in Axis 2 (VA2)	2.66 ± 0.12	2.55 ± 0.19	** < 0.001**	0.149	1.93 ± 0.43	** < 0.001**			
Variation in Axis 3 (VA3)	2.63 ± 0.13	2.56 ± 0.17	** < 0.001**	0.920	1.94 ± 0.42	** < 0.001**			

Bold text identifies all p values that are p < 0.05

^a^After fitting a linear model to correct for observed differences in age and sex between patients with MND and controls

^b^Comparison of patients with MND that contributed hip- vs. wrist-based actigraphy. Data presented as median ± interquartile range, or *n* (%). Medians compared using Wilcoxon rank-sum tests. Proportions compared using Chi-squared tests

### Anthropometric and clinical measures

The clinical history of each participant was noted, and for patients with MND, the ALS Functional Rating Scale-Revised (ALSFRS-R) [9] was implemented by a healthcare professional. The ALSFRS-R was summarised into four domains: bulbar (questions 1–3), fine motor (questions 4–6), gross motor (questions 7–9), and respiratory (questions 10–12) domains [10]. The King’s stage was derived from the ALSFRS-R [11]. The slope of the ALSFRS-R with respect to time (ΔFRS) was determined as [ΔFRS = (ALSFRS-R score − 48)/months since symptom onset].

### Data collection and statistical analyses

For the wrist-based cohort, patients wore a *GT9X* accelerometer on their non-dominant wrist for eight continuous days, beginning from 11:59AM (AEST—UTC + 10) local time of the day the device was given. Patients were instructed to wear the device for the duration of the recording period and notified the research team if they were not able to complete the collection period due to discomfort or recording failure. Accelerations alongside the vertical (axis 1), sideway (axis 2), and forward (axis 3) axes were recorded at 30 Hz. ActiLife software (ver 6.13.4) was used to download ActiGraph “count” summaries in each axis with an epoch of 10 s. The “low frequency extension” option was selected to increase sensitivity for movement in this cohort. Wear time was identified using the Choi algorithm [12, 13] and sleep periods were identified by applying the Cole–Kripke algorithm [14]. The epochs that were then identified as non-wear time, or sleeping epochs were excluded from this analysis.

As done previously [4], three features were extracted from the count data and summarised over each day. Proportion of time spent active (PA) was defined as the proportion of epochs with a score above 100 counts/min (below which is commonly considered a threshold for “sedentary” behaviour [15]). The overall degree of movement was assessed as the product of the mean and standard deviation of the natural log-transformed vector magnitude between the three axes [VM = mean × standard deviation log((*x*^2^ + *y*^2^ + *z*^2^)^1/2^ + 1)], representing a trade-off between participants’ ability for both large movements (standard deviation), and frequent movements (mean). The capacity for movement was assessed as the variability of the log-transformed counts within the epoch between each axis [VA1 – 3 = log(counts within axis + 1)].

For hip-based actigraphy, axis 1 corresponds to the superior orientation (in line with gravity). For wrist-based actigraphy (at anatomical positioning), axis 1 corresponds to the medial orientation; however, true orientation is highly affected by wrist positioning. Summarised counts were averaged over 24-h periods, starting at midnight local time. The mean of the daily summaries over each recording period was used for the statistical inclusion.

Data were reported as median ± interquartile range, or *n* (%). Numerical data were compared using Wilcoxon rank-sum tests, and correlations were assessed using Kendall’s tau-B. Categorical proportions were compared using Fisher’s exact tests. Linear mixed-effects models were fitted to assess the performance of activity measures for monitoring loss of function in patients with MND; coefficients were reported as estimate ± standard error [95% confidence interval], and significance was estimated using Satterthwaite’s t tests. All data analysis and presentation were prepared in RStudio (ver 1.4.1717) running R (ver 3.6.3), with the tidyverse (ver 1.3.1), lme4 (ver 1.1.27), and ggpubr (ver 0.4.0) packages.

## Results

### Cross-sectional case–control comparison

**Fig. 2:**
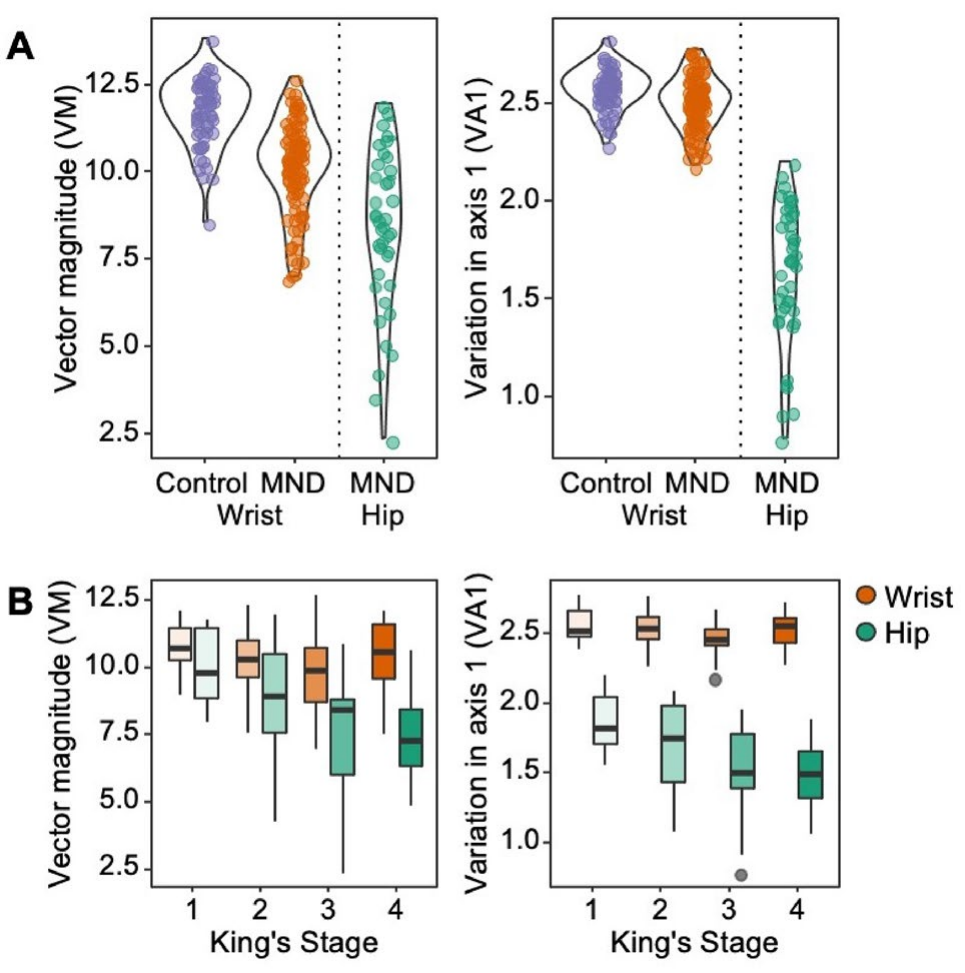
Cross-sectional activity measures. **A** Patients with MND had lower activity summaries measured from the wrist (orange), as compared to controls (blue) (*p < *0.001). Patients wearing hip-worn trackers (green) measured lower activity summary scores than wrist-worn trackers (orange) (*p < *0.001). **B** Patients wearing hip-worn activity trackers reported activity summaries declined alongside disease severity, as reported by King’s staging; however, this relationship was not observed in wrist-worn trackers

Case–control comparisons were completed for wrist-based actigraphy only and thus limited to the Australian cohort of patients and controls. Demographic features between cases and controls are presented in Table 1; there was a higher proportion of males in the patient cohort (77% vs 47%, *p < *0.001), and the control cohort was older (55.78 vs 59.90 years, *p = *0.001). We observed decreased activity across all measures in patients with MND, relative to controls (Fig. 2A, Table 1, all *p < *0.001). The differences in PA and VM persisted after fitting a linear model to correct for age and sex, however, after adjusting VA1-3 were similar between the groups (Table 1).

### Cross-sectional within-case comparisons

Comparing clinical features between wrist- (Australia) and hip-based (The Netherlands) actigraphy cross-sectional cohorts, we observed differences in BMI (26.54 vs 24.20 kg/m^−2^, *p = *0.013) and riluzole use; patients from Australia were less likely to use riluzole (50% vs 75%, *p = *0.008; Table 1). The ALSFRS-R total scores, ALSFRS-R sub-domain scores, King’s Staging, and ΔFRS were similar between patient cohorts. All measures of activity were increased in patients with wrist-based actigraphy when compared to patients with hip-based actigraphy (Fig. 2A, Table 1, all *p < *0.001). Only measures derived from hip-based actigraphy declined alongside disease progression, as assessed using King’s staging (Fig. 2B).

### Longitudinal within-case comparisons

**Fig. 3:**
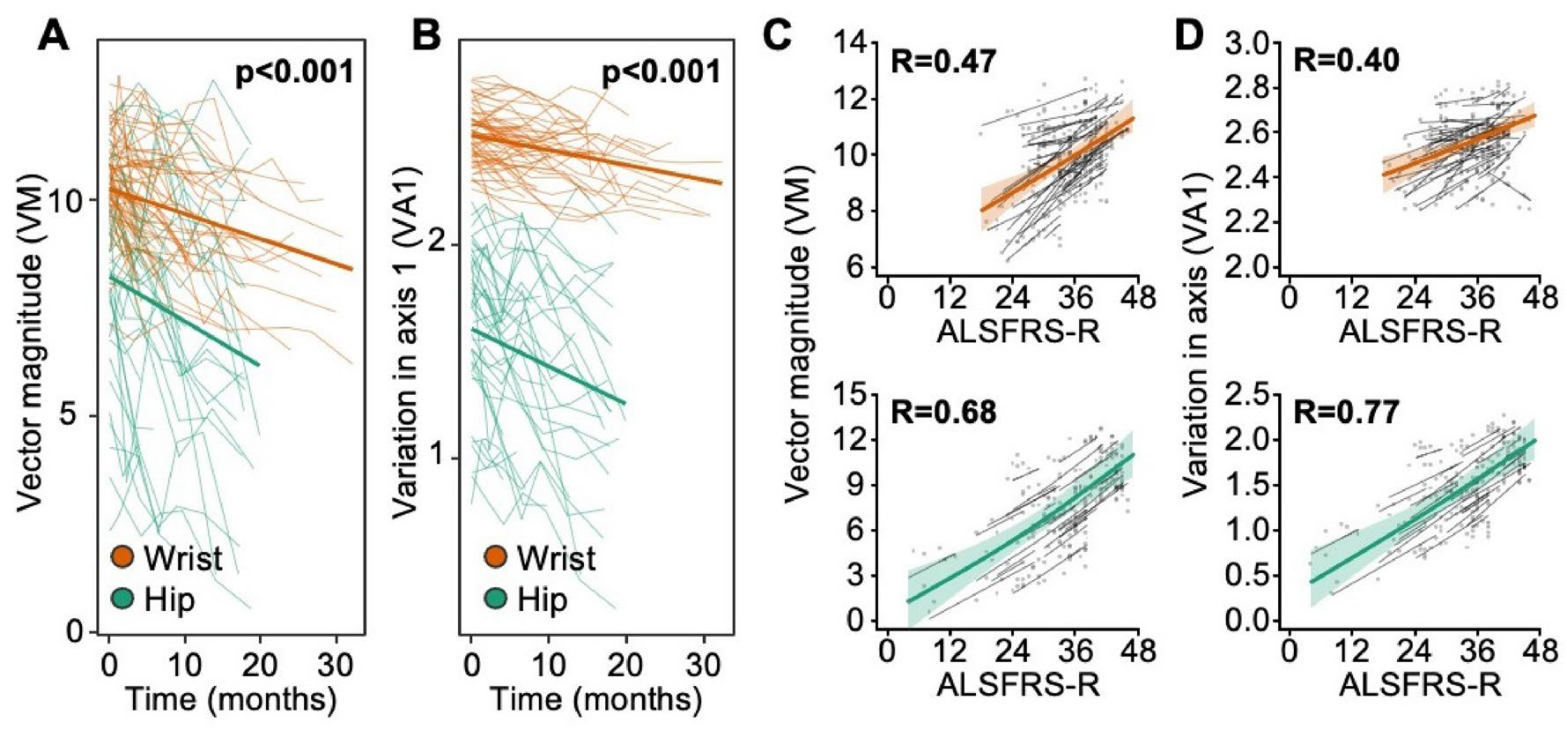
Longitudinal outcomes. **A**, **B** Faint lines indicate individual patient activity summary scores since first assessment. Bolded lines were derived from a mixed-effects linear regression, with a random intercept and slope per patient. Hip-worn trackers had a wider range of scores and reported lower overall scores than wrist-worn trackers. Activity scores declined over time (all *p < *0.001). **C**, **D** Plots show the relationship between ALSFRS-R and activity scores per patient between repeat measures. Coloured lines represent fixed effect component of model; grey lines represent individual subject trajectories. Lines were derived from a mixed-effects linear regression, using restricted cubic splines to fit a curve between variables of interest (participant was treated as a random effect). 95% confidence intervals (shaded region) were estimated using bootstrapping. Correlations were estimated by scaling and centring the variables before fitting the linear regression (all *p < *0.001)

As a group, all outcomes declined over time, although considerable between-subject variability was observed (Fig. 3A, B, Table 2). Repeat-measure regressions between ALSFRS-R scores and actigraphy outcomes (Fig. 3C, D) are reported in Table 3.

**Table 2:** Activity outcomes decline over time in both wrist- and hip-worn accelerometers

	Wrist-based actigraphy (*n = *97)				Hip-based actigraphy (*n = *42)			
	Intercept	Slope (month^−1^)	95% CI	*p*	Intercept	Slope (month^−1^)	95% CI	*p*
ALSFRS-R								
Total	37.7	− 0.60	[− 0.75, − 0.47]	** < 0.001**	36.4	− 0.62	[− 0.80, − 0.44]	** < 0.001**
Fine	9.0	− 0.17	[− 0.22, − 0.13]	** < 0.001**	7.7	− 0.22	[− 0.30, − 0.13]	**< 0.001**
Gross	7.7	− 0.14	[− 0.18, − 0.09]	** < 0.001**	7.4	− 0.18	[− 0.25, − 0.11]	**< 0.001**
Actigraphy outcomes								
Proportion active (PA)	0.644	− 0.002	[− 0.004, − 0.007]	**0.005**	0.276	− 0.006	[− 0.008, − 0.004]	**< 0.001**
Vector magnitude (VM)	10.2	− 0.08	[− 0.11, − 0.06]	** < 0.001**	8.45	− 0.17	[− 0.23, − 0.12]	**< 0.001**
Variation in Axis 1 (VA1)	2.51	− 0.007	[− 0.009, − 0.005]	**< 0.001**	1.64	− 0.027	[− 0.036, − 0.019]	** < 0.001**
Variation in Axis 2 (VA2)	2.52	− 0.010	[− 0.013, − 0.007]	** < 0.001**	1.85	− 0.028	[− 0.038, − 0.019]	** < 0.001**
Variation in Axis 3 (VA3)	2.55	− 0.006	[− 0.009, − 0.004]	** < 0.001**	1.89	− 0.026	[− 0.035, − 0.018]	**< 0.001**

Bold text identifies all p values that are p < 0.05

Monthly changes in ALSFRS-R and actigraphy outcomes were estimated by fitting a linear mixed-effects model with a per-patient slope and intercept for each measure over time

**Table 3:** Linear associations between measures of activity and ALSFRS-R over time

	Predictor				
	PA	VM	VA1	VA2	VA3
Wrist-based actigraphy (*n = *97)					
ALSFRS-R	24.58 [14.67, 34.45]	2.66 [2.17, 3.14]	21.93 [16.78, 27.02]	21.74 [17.81, 25.61]	20.88 [15.73, 25.98]
Fine (Q4-6)	11.81 [7.92, 15.73]	1.16 [0.98, 1.34]	9.96 [8.07, 11.85]	9.25 [7.81, 10.69]	9.18 [7.25, 11.10]
Gross (Q7-9)	7.818 [4.12, 11.53]	0.85 [0.67, 1.03]	7.78 [5.93, 9.61]	7.2 [5.78, 8.62]	7.21 [5.36, 9.05]
Hip-based Actigraphy (*n = *42)					
ALSFRS-R	35.21 [27.21, 43.22]	1.72 [1.41, 2.03]	12.46 [10.57, 14.35]	11.97 [10.05, 13.89]	10.24 [8.30, 12.19]
Fine (Q4-6)	11.04 [8.13, 13.99]	0.55 [0.44, 0.67]	3.81 [3.09, 4.54]	3.59 [2.86, 4.34]	3.14 [2.42, 3.87]
Gross (Q7-9)	12.27 [9.45, 15.13]	0.61 [0.50, 0.73]	4.31 [3.60, 5.03]	4.03 [3.31, 4.77]	3.52 [2.82, 4.25]

Longitudinal correlations were estimated by fitting linear mixed-effects regressions over measures collected from all patients with MND, with a per-patient slope and intercept estimated. All models *p < *000.1

To model the efficacy for each activity measure to inform future functional decline, linear mixed-effects models were fitted for the ALSFRS-R (and each motor domain) (Table 4). For hip-based measures, axes 1 (tau = 0.25, *p = *0.020) and 3 (tau = 0.22, *p = *0.041) were found to be correlated with future declines in total ALSFRS-R. For wrist-based measures, axes 2 and 3 were found to be correlated with future declines in total ALSFRS-R. Moreover, most wrist-based activity summaries (all but PA) correlated with future declines in the “fine motor” domain of the ALSFRS-R (taus 0.14–0.23). Similar outcomes were not observed for hip-based actigraphy measures.

**Table 4:** Correlations between activity measures and future changes in patients' ALSFRS-R

	ALSFRS-R (Total)		ALSFRS-R (Fine)		ALSFRS-R (Gross)	
	Tau	*p*	Tau	*p*	Tau	*p*
Wrist-based actigraphy (*n = *94)						
Proportion active (PA)	− 0.06	0.414	0.09	0.212	0.00	0.966
Vector magnitude (VM)	0.07	0.296	0.19	**0.006**	− 0.07	0.328
Variation in axis 1 (VA1)	0.13	0.056	0.14	**0.049**	− 0.09	0.236
Variation in axis 2 (VA2)	0.14	**0.040**	0.23	**0.001**	− 0.10	0.170
Variation in axis 3 (VA3)	0.15	**0.034**	0.17	**0.017**	− 0.08	0.236
Hip-based actigraphy (*n = *42)						
Proportion active (PA)	0.14	0.210	0.06	0.605	0.09	0.400
Vector magnitude (VM)	0.21	0.060	0.10	0.342	0.13	0.234
Variation in axis 1 (VA1)	0.25	**0.020**	0.16	0.135	0.18	0.090
Variation in axis 2 (VA2)	0.18	0.095	0.07	0.532	0.12	0.280
Variation in axis 3 (VA3)	0.22	**0.041**	0.15	0.153	0.14	0.190

Bold text identifies all p values that are p < 0.05

Linear mixed-effects regressions were fit to estimate the individual random slopes and intercepts for each activity measure. For each measure, a Kendall tau-B correlation was estimated comparing the patients' baseline activity and the longitudinal slopes of the ALSFRS-R

## Discussion

There are few sensitive, specific, clinical markers available for monitoring progression of MND. As such, trial design and routine care are reliant on patients travelling to central multidisciplinary clinics for basic care. Collection of patient outcomes outside of the traditional clinical setting may offer improved capacity to monitor disease progression while simultaneously lowering burden of care on patients. We investigated the utility of at-home wrist-based actigraphy as measures of functional decline in patients with MND and contrasted outcomes with previously validated hip-based measures [4]. As with hip-based actigraphy, wrist-based measures show potential to monitor functional change; however, our results suggest that device placement will greatly influence outcomes.

We report lower wrist-based activity scores in patients when compared to controls. Patients had a lower degree of movement and lower proportion of time spent active. This suggests that the loss of physical function in patients with MND can be detected with wrist-based actigraphy. However, the overall capacity for movement in patients (i.e. the variation in axes) was not different from controls, and so not all measures may be informative. This is not unexpected, given that validation of actigraphy in other cohorts of patients with neurodegenerative disease show similar outcomes; a recent report in patients with Huntington’s disease showed that patients did not have fewer step counts or activity bouts, but did have increase within-bout variability [16].

Measures of actigraphy are not intended as a diagnostic tool; rather, it is hoped that outcomes will provide information on functional decline. We observe declines in activity scores alongside some ALSFRS-R subscores. Specifically, wrist-based actigraphy measures consistently correlated with functional loss in the “fine-motor” domain of the ALSFRS-R; changes within this domain are highly dependent on upper-limb mobility [9]. Weaker associations were observed in the gross motor domain, suggesting reduced capacity for current wrist-based actigraphy measures to infer change in general functional decline. By comparison, hip-based actigraphy measures are strongly associated with change in gross motor function, as previously observed [4]. This is a logical outcome, given the impact of wear location on the collection of information that might be used to infer change in fine and gross movements. To improve understanding of the impact of device positioning, future studies should contrast outcomes between different wear locations on the same individual, during the same period.

Results from this study suggest that device-specific outcome measures (e.g. automated algorithms, summary measures) must be carefully selected to match wear location, and investigators need to accept a higher degree of variability when adopting wrist-based actigraphy. Higher activity scores using wrist-based actigraphy matches the more dynamic use of the wrist compared to the hip, which may introduce additional variance. Critically, increasing gross disability may not imply declines in upper-limb/wrist use, as people may increase use of upper limbs to compensate for declines in gross motor function. A criticism of our finding, however, is lack of inclusion of patients with high degrees of disability in the wrist-based actigraphy cohort. Study outcomes are contrasted against the ALSFRS-R; in the wrist-based cohort, the lowest ALSFRS-R score is 18 points, whereas the lowest ALSFRS-R score in the hip-based actigraphy cohort is 4 points. This is best appreciated when considering data presented in Fig. 2. As such, current results do not fully address the utility of wrist-based actigraphy in patients that experience a dramatic decline in functional capacity.

Compensation of worsening lower-limb dysfunction through increased upper-limb activity is apparent in repeat measures for some patients with wrist-based actigraphy. As such, declines in the ALSFRS-R were not consistently correlated with changes in wrist-based measures. While most patients presented with a positive association between the activity measure and their functional capacity (as inferred from the ALSFRS-R), a portion of patients produced negative associations. Patients with a neuromotor disease, such as MND, are not representative of a typical training dataset, and as such, assessments of their movements and behaviours may be less accurate. For example, a recent study using actigraphy to classify activity in an aged cohort found that wrist-worn devices were able to reasonably classify activity thresholds, but underestimated step counts by about 30% – a concern for the implementation of actigraphy as a clinical endpoint [16]. These findings point to the need for improved design of actigraphy endpoints in MND research and care that better reflect patients’ physical capability.

Of interest, we found that decreased activity scores were detected in the wrist before patients reported reduced function in fine-motor skills (as inferred through a change in the fine-motor domain subscores of the ALSFRS-R). This outcome agrees with the common hypothesis that spread in MND (particularly in the spine) generally occurs within an affected region before moving to adjacent regions [17, 18] and may infer potential use for limb-based actigraphy as an early, low-burden, marker for predicting the spread of disability in affected regions before this is apparent to the patient. To better understand the variable impact of device positioning, and the implications for biomarker development, it would be prudent to assess the impact of multiple device positions on the individual and their relation to various activity metrics and differing disease presentations.

There is widespread interest in the development of improved methods for routine monitoring of patients outside a traditional clinical setting [4, 19–21] (see also NCT05276349). While allowing for improved patient monitoring [3], collection of at-home measures has the advantage of improving the veracity of assessments by capturing patients’ natural behaviours. Results from this study suggest that wrist-based actigraphy can be used to infer functional decline in patients with MND, although the differing performance compared to the hip underscores the need to select device wear location based on outcome of interest. The popularity of wrist-based actigraphy has increased considerably alongside the widespread use of smartwatches [22], and with this, we have seen an evolution of additional wrist-based measures that might be informative. As such, there is impetus to establish best-practice for the use of actigraphy in neurodegenerative diseases, such as MND. Further refinements in establishing wrist-based actigraphy as a technique for monitoring functional decline in MND will enable robust multi-domain remote patient monitoring.

## Data Availability

Anonymised data will be made available or shared upon reasonable request.
